# Whole β-glucan particle attenuates AOM/DSS-induced colorectal tumorigenesis in mice *via* inhibition of intestinal inflammation

**DOI:** 10.3389/fphar.2023.1017475

**Published:** 2023-01-12

**Authors:** Yewen Xie, Fang Shao, Xuehan Duan, Jun Ding, Yongling Ning, Xiao Sun, Lei Xia, Jie Pan, Jie Chen, Shuyan He, Dong Shen, Chunjian Qi

**Affiliations:** ^1^ Medical Research Center, The Affiliated Changzhou Second People’s Hospital of Nanjing Medical University, Changzhou, China; ^2^ Oncology Institute, The Affiliated Changzhou Second People’s Hospital of Nanjing Medical University, Changzhou, China; ^3^ Department of Oncology, The Affiliated Jiangyin Hospital of Nantong University, Jiangyin, Jiangsu, China

**Keywords:** yeast β-glucan, colitis-associated colorectal cancer, chronic colitis, dendritic cell, intestinal inflammation

## Abstract

Yeast β-glucan is a polysaccharide purified from *the Saccharomyces cerevisiae* cell wall, and its multiple biological activities are essential for immune regulation. However, the effect of β-glucan on the intestinal immune response during colitis-associated colorectal cancer (CAC) is unclear. Here, we explore the possible role of β-glucan in the development of CAC. Wild type (WT) mice with CAC induced by azoxmethane (AOM) and dextran sodium sulfate (DSS) had fewer tumors than untreated mice after oral β-glucan because of increased antitumor dendritic cells (DCs) in the tumor microenvironment, resulting in more CD8^+^ T cells and the production of related cytokines. β-glucan also increased resistance to DSS-induced chronic colitis by reshaping the inflammatory microenvironment. These data suggest that β-glucan improves experimental intestinal inflammation and delays the development of CAC. Therefore, β-glucan is feasible for treating chronic colitis and CAC in clinical practice.

## Introduction

Colorectal cancer (CRC) is a common malignant tumor in the digestive tract. Its incidence and mortality rate rank 4th and 2nd among malignant tumors, respectively, and the 2nd most common tumor among women (Wolf et al., 2018; Dekker et al., 2019). The clinical treatment options for CRC include surgery, chemotherapy, radiotherapy, and targeted therapy. Despite current advances and improvements in the early screening and therapy of CRC, the overall survival rates of CRC patients are discouragingly low (Siegel et al., 2019). CRC is closely associated with chronic intestinal inflammation, most commonly inflammatory bowel disease (IBD), mainly including ulcerative colitis (UC) and Crohn’s disease (CD) (Tang et al., 2021). The functional relationship between inflammation and cancer was first proposed by Rudolf Virchow in 1863 (Balkwill and Mantovani, 2001), and subsequently, cancer-associated inflammation (CRI) was considered a marker of cancer (Colotta et al., 2009).

Colitis-associated colorectal cancer (CAC) is a special type of colorectal cancer. Epidemiological studies have shown that the risk of CAC in IBD patients is 2–4 times that of the normal population, and approximately 20% of IBD patients will develop CAC in the next 10 years (Gaidos and Bickston, 2016). During the development of inflammation-associated cancers, the interaction between epithelial cells that harbor mutations in tumor suppressor genes or proto-oncogenes, disordered immune cells and the microenvironment enriched in pro-inflammatory mediators is conducive to tumor growth (Quail and Joyce, 2013). Mediators such as IL-6 activate STAT3 signaling to promote epithelial cell proliferation and induce inflammatory tumor formation (Ye et al., 2019). Additionally, IL-10 produced by CD4^+^ T cells promotes tumors by inhibiting T-cell functions and the upstream activities of antigen presenting cells (APCs) (Barilla et al., 2019). In this case, the recruitment of innate immune cells by chemokines such as CXCL1 or CXCL2 further contributes to exacerbating the native environment (Chen et al., 2020). From these data, it is clear that a better knowledge of the pathways implicated in the expression of pro-inflammatory genes in intestinal immune cells could help to design new strategies to restrain inflammation-associated malignancies.

Whole glucan particles (WGP), a natural polysaccharide, are commonly located in the cell walls of plants, fungi and algae. The biological activity of β-glucan, including its antitumor and anti-infective effects, has been extensively investigated and is dependent on various sources and structures (Qi et al., 2011; Deng et al., 2012). Studies have indicated that only yeast-derived β-glucans, which possess a β-1,3-D-glucan backbone grafted with long β-1,6-glucan side chains, behave as immunomodulators (Ensley et al., 1994). The immune function of yeast-derived β-glucans is mainly attributed to β-1,3-D-glycoside, a ligand of the dectin-1 receptor, through which β-glucans can be recognized by immune cells expressing dectin-1, such as DCs (Plato et al., 2013). In murine tumor models, we found that orally administered WGP elicited potent antitumor immune responses and drastically downregulated immunosuppressive cells, leading to delayed tumor progression. Deficiency of the dectin-1 receptor completely abrogated WGP-mediated antitumor effects (Li et al., 2010; He et al., 2022). Deficiency of the dectin-1 receptor completely abrogated β-glucan mediated antitumor effects (Qi et al., 2011). Herein, an insoluble whole β-glucan particle (WGP) was used as a novel immunotherapy for CAC in this study.

Based on these previous findings, we investigated the possible role of WGP in the development of colitis and colitis-associated tumorigenesis. Our data suggest that WGP may inhibit the occurrence and progression of colon tumors by inhibiting intestinal inflammation, but the specific mechanism needs to be further studied.

## Materials and methods

### Materials

WGP was derived from the cell walls of *Saccharomyces cerevisiae*. In a series of basic and acidic extractions, the cytoplasm and other cell walls such as mannose and other polysaccharides were removed and left intact to give β-1, 3-glucan shells. To eliminate any trace amounts of LPS contamination, WGP was suspended in 200 mM NaOH for 20 min at room temperature (RT), washed thoroughly and resuspended in LPS-free water as described previously (Qi et al., 2011). The endotoxin level was .06 EU/mL as tested by the gel-clot method (Associates of Cape Cod, East Falmouth, MA, United States).

### Mice

Female SPF C57BL/6 mice and Dectin-1^−/−^ mice aged 6–8 weeks were purchased from Changzhou Cavens Company. All mice were maintained under specific pathogen-free conditions. The study protocol was approved by the Nanjing Medical University Animal Care and Use Committee.

### Establishment of CAC model

Modeling and analysis of colitis-associated tumorigenesis was performed according to a previously reported protocol (Neufert et al., 2007). Wild type (WT) and Dectin-1^−/−^ mice were injected intraperitoneally with azoxmethane (AOM; 12.5 mg/kg; Sigma‒Aldrich, Shanghai, China) on Day 0 and then maintained on regular water and diet for 7 days. After that, the mice were fed 2% dextran sulfate sodium (DSS; molecular mass 36,000–50,000 Da, MP Biomedicals, Beijing, China) in their drinking water for 7 days and then received normal water for 14 days. This cycle was repeated three times in therapy. WGP was dissolved in sterile water and orally administered to the mice (10 mg/mL) from the first day until the 10th day. Control mice were treated with water alone. Body weight was measured every day. We used the DAI to quantify colitis severity as previously described (Rabbi et al., 2014). After induction of tumorigenesis, mice were euthanized on Day 84. All colon tissues, tumor tissues, serum, spleens and MLNs were collected on Day 84. Upon necropsy, the numbers and size of visual polyps in colons were measured using a digital caliper. We also monitored the survival rate when the mice received 2% DSS to induce tumors in the indicated groups within 84 days.

### Isolation of cLP and tumor cells

Colonic lamina propria (cLP) cells and colon tumors were isolated from the study mice as described previously (Landers et al., 2002). Briefly, colons were isolated, resected, opened longitudinally, washed, and the middle and lower 1/3 section of the colorectal was cut into small pieces smaller than 1 mm^3^, followed by digestion medium consisting of RPM1640, 5% fetal bovine serum (FBS) with collagenase type IV, hyaluronidase, and deoxyribonuclease (Sigma‒Aldrich, Shanghai, China) for 30 min at 37°C on a rotating platform. Samples were then filtered through a 70 μm cell strainer and washed twice with phosphate-buffered saline (PBS, Thermo Fisher Scientific Inc.) by 2000 rpm centrifugation. Finally, the cLP cells suspension were obtained. Colon tumor cells were prepared in the same way. The tumor generated on the colorectal was dissected and then cut into small pieces less than 1 mm^3^, then, digested at 37° with digestive enzyme mixture for 30 min and vortexed every 5 min. Finally, the sample was filtered with a 70 m cell filter, and the tumor cell suspension was obtained after centrifugation twice with 2,000 RPM in PBS.

### Histological analysis

Briefly, a 1 cm segment of the distal colon was immediately fixed with 4% paraformaldehyde and embedded in paraffin. After sectioning into 4 µm sections, colon segments were stained with H&E to assess tissue integrity. The degree of colitis was assessed as previously described (Kim et al., 2012):degree of inflammatory cell infiltration (normal, 0–dense inflammatory infiltrate, 3), crypt architecture (normal, 0–severe crypt distortion with loss of entire crypts, 3), crypt abscess (absent, 0–present, 1), goblet cell depletion (absent, 0–present, 1), and muscle thickening (base of crypt sits on the muscularis mucosae, 0–marked muscle thickening, 3). The total histologic score was derived by summing each individual score. Tumor was assessed by a pathologist blinded to the mouse genotype and treatment using clinical and pathological scores as described previously (Kargl et al., 2013; Wirtz et al., 2017).

### Flow cytometry

Surface and intracellular molecule staining was performed as previously described (Ning et al., 2018). Cells were resuspended in staining buffer, blocked with an Fc-blocking monoclonal antibody for 15 min on ice, and stained with fluorescently labeled antibodies against CD45, CD11c, CD11b, F4/80, LY-6G, MHC class I, MHC class II, CD3, CD4, or CD8 (Biolegend, San Diego, CA) on ice for 30 min. After a washing step, flow cytometry was performed on a BD FACS Canto II (San Jose, California, United States). Mouse serum cytokines were detected with a Mouse Th Cytokine Flow Assay Kit (Biolegend, LEGENDplex™, NO.741053) according to the manufacturer’s protocol. Flow cytometry data were analyzed using Flow Jo software V8 (Tree Star, New Jersey, United States).

### Induction of intestinal chronic inflammation

On day 0, the mice were divided into groups and fed with water containing 2% DSS directly for 7 days, followed by normal water supply for 14 days. This was a cycle with three repeated cycles as the CAC model administration plan. During the modeling period, mice in the experimental group were treated with oral WGP from the beginning of each cycle to day 10, while mice in the control group were treated with water only. The body weight was measured and recorded daily, the blood in the stool was observed, and the disease activity index was recorded. At the same time, the survival rate during the inflammatory process induced by 2% DSS drinking water was monitored. On day 84, blood was collected from the orbit to collect serum, and the mice were euthanized and all relevant tissue samples including colonic tissue, spleen and mesenteric lymph nodes were collected.

### RNA extraction and quantitative RT‒PCR analysis

Total RNA of distal colonic tissues was extracted using TRIzol (Invitrogen, Thermo Fisher Scientific, CN) according to the manufacturer’s instructions. High-fidelity cDNA was generated from each RNA sample with a cDNA Reverse Transcription Kit (Applied Biosystems, CN). Quantitative reverse transcriptase-PCR was performed using a SYBR Kit (Invitrogen, Thermo Fisher Scientific, CN) according to the manufacturer’s protocol. Data were acquired on an ABI ViiA 7 Real-time PCR system. We used the 2^−ΔΔCt^ quantification method with mouse glyceraldehyde 3-phosphate dehydrogenase (GAPDH) as an endogenous control. The primer sequences designed with Primer Express Software Version 2.0 (Applied Biosystems) are summarized in Supplementary Table S1. Data were acquired on an ABI ViiA 7 Real-time PCR system.

### Statistical analysis

All experiments were independently performed three times. Data are presented as the mean ± SEM. The data were analyzed using an unpaired Student’s *t*-test to determine the significance of differences between two groups. The log-rank test (Mantel-Cox) was used to evaluate survival differences. Statistical analysis was performed in GraphPad Prism eight software. The threshold for statistical significance was *p* < .05. No statistical methods were used to predetermine the sample size.

## Results

### WGP alleviated AOM/DSS-induced colorectal tumorigenesis in WT mice

To investigate the role of WGP in colitis-associated tumorigenesis, we used the combination of the mutagen AOM and DSS to establish CAC mouse model (Neufert et al., 2007). This model simulates the whole process from normal colorectal mucosa to inflammation and then to tumor formation. Its pathological characteristics are similar to those of human colorectal cancer, and it can roughly reflect the process of human colon inflammation in tumors, which is a generally accepted method at present (Ritter and Greten, 2019).

**FIGURE 1:**
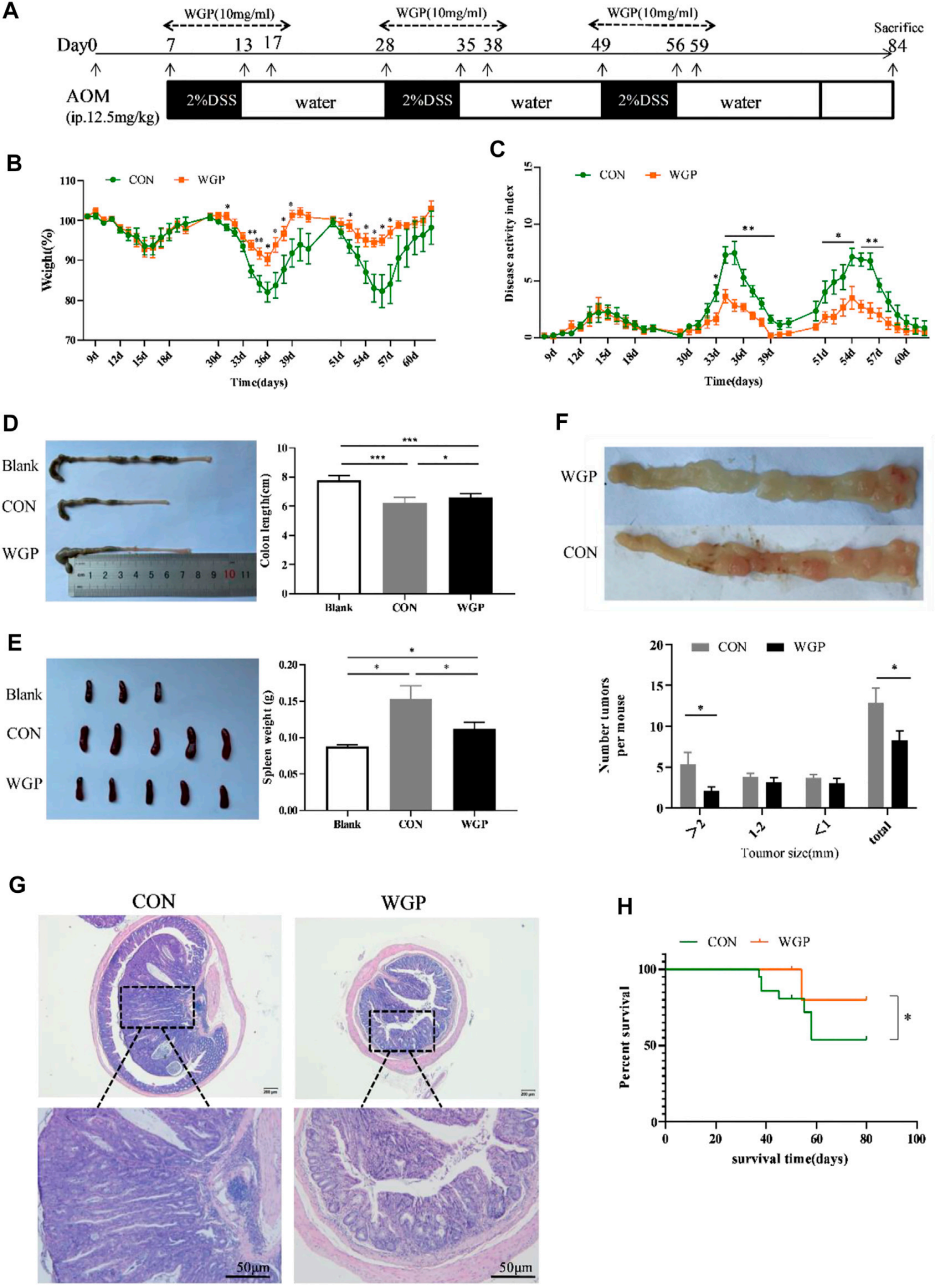
WGP treatment can attenuate colitis-associated colorectal cancer (CAC). **(A)** Induction procedure for AOM/DSS-induced CAC in C57BL/6 mice and WGP administration. **(B–C)** Body weight **(B)** and disease activity index during each treatment cycle **(C)** of mice that received 2% DSS-containing water (CON group, green dotted line) or 2% DSS combined with WGP orally (WGP group, orange dotted line). For statistical comparisons, an asterisk indicates CON vs. WGP. **(D–F)** Colon length **(D)**, spleen weight **(E)**, and the number of tumor representative pictures of colons and the size distribution of colorectal tumors **(F)** at Day 84 in AOM/DSS-induced mice treated with or without WGP. **(G)** Representative H&E stained images and corresponding tumor area of distal colon cross-sections. **(H)** Survival curves of mice treated with water or WGP, *p < .05 by log-rank test. Data are representative of three independent experiments with *n* = 8–10 mice per group. The statistical significance of differences was determined by multiple t tests **(B,C,F)** and one-way ANOVA **(D,E)**. *p < .05, **p < .01, ***p < .001. The results are presented as the means ± SEMs.

WT mice were treated with AOM and DSS as indicated in Figure 1A. Compared to the CON group, we found that preventive WGP treatment significantly attenuated weight loss caused by DSS (Figure 1B). The disease activity index (DAI), a composite score used to evaluate the clinical manifestations of colonic inflammation, drastically decreased upon WGP treatment compared to the CON group (Figure 1C). Colon length is a common indicator of colon injury, and we observed significantly longer colons in WGP-treated mice than in CON mice following DSS exposure (Figure 1D), and the spleen weight of WGP-treated mice was lighter than that of CON mice (Figure 1E).

At the end of the experiment, we observed the formation of multiple tumor nodules in the middle and lower colorectal segments of mice. Colons of WGP-treated mice exhibited a decreased number of tumors in the distal region that were also smaller in size than those of the water-treated mice (Figure 1F). In addition, histological analysis of colonic lesions in water-treated mice revealed obviously increased tumor areas and ahigh degree of tissue atypia. In contrast, WGP treatment markedly reduced these pathological changes, with a well-preserved mucosal architecture, limited tumor area, and low-grade tumor tissue atypia (Figure 1G). Furthermore, during the formation of colitis-associated tumors, the survival time of WGP-treated mice was significantly prolonged (Figure 1H). These results suggest that WGP can alleviate AOM/DSS-induced CAC.

Since the immune effect of WGP is mainly mediated by its pattern recognition receptor dectin-1 (Carvalho et al., 2012), to further prove the importance of WGP in dectin-1 recognition, we used dectin-1-deficient mice to induce CAC under the same conditions. The data showed that the effect of WGP was eliminated in dectin-1^−/−^ mice. All clinical features, such as weight loss, DAI score, colon length, spleen weight, the degree of terminal colon tumor formation and tissue atypia, did not differ significantly between the two groups (Supplementary Figure S1), further confirming that WGP alleviated CAC to play an antitumor role dependent on Dectin-1.

### WGP regulates the infiltration of immune cells in WT mice with CAC

**FIGURE 2:**
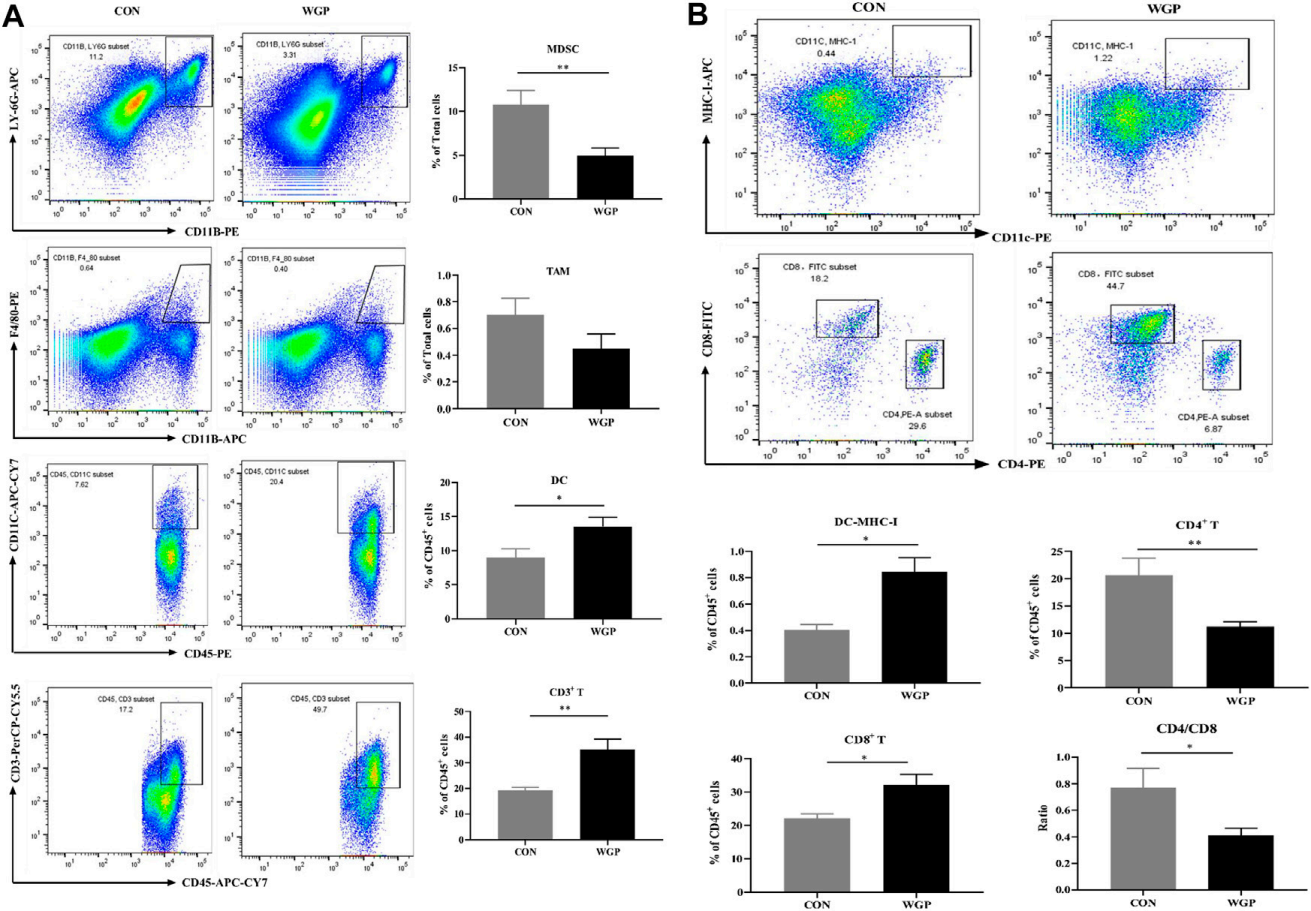
WGP alters immune cell infiltration in CAC **(A)** At Day 84, the representative staining, percentage of MDSCs (CD11b^+^ LY-6G^+^), macrophages (CD11b^+^ F4/80^+^) gated in total cells, dendritic cells (DCs) (CD45^+^ CD11c^+^), and T cells (CD45^+^ CD3^+^) gated in CD45^+^ cells of the tumor were determined by flow cytometry. **(B)** MHC-I^+^ DCs gated in DCs, CD8^+^ T cells and CD4^+^ T cells gated in CD3^+^ T cells were determined by flow cytometry. Numbers adjacent to the outlined areas indicate the percentage of the gated population in each group (*n* = 5–6). *p < .05, **p < .01 vs. CON by unpaired Student’s t-test.

Immune cells play an important role in the tumor microenvironment; thus, we evaluated the degree of immune infiltration in the tumors. The CD45^+^ cells of these mice were quantified. We observed a decrease in the percentage of CD11b^+^ LY-6G^+^ Myeloid-derived suppressor cell (MDSCs), a significant increase in CD11c^+^ DC and CD3^+^ T cells, but no significant change in CD11b^+^ F4/80^+^ macrophages in the tumor microenvironment of mice treated with WGP compared with CON (Figure 2A). A greater proportion of MHC-I^+^ expressing DCs in the increased CD11c^+^ DC cells further induced a marked increase in CD8^+^ T cells, however, the CD4/CD8 ratio decreased due to a marked decrease in CD4^+^ T cells (Figure 2B). Analysis of immune cells in the tumor microenvironment of dectin-1^−/−^ mice showed that WGP did not increase the proportion of DCs and T cells, but still reduced MDSCs (Supplementary Figure S2). Although the data suggest that MDSCs reduced in the tumor microenvironment of dectin-1^−/−^ mice, this response appears to be redundant because WGP did not affect colon tumor formation in dectin-1^−/−^ mice. These results suggest that WGP alleviates CAC by reshaping immune cell infiltration in the tumor microenvironment, especially DCs.

### WGP regulates the expression of related cytokines in CAC

**FIGURE 3:**
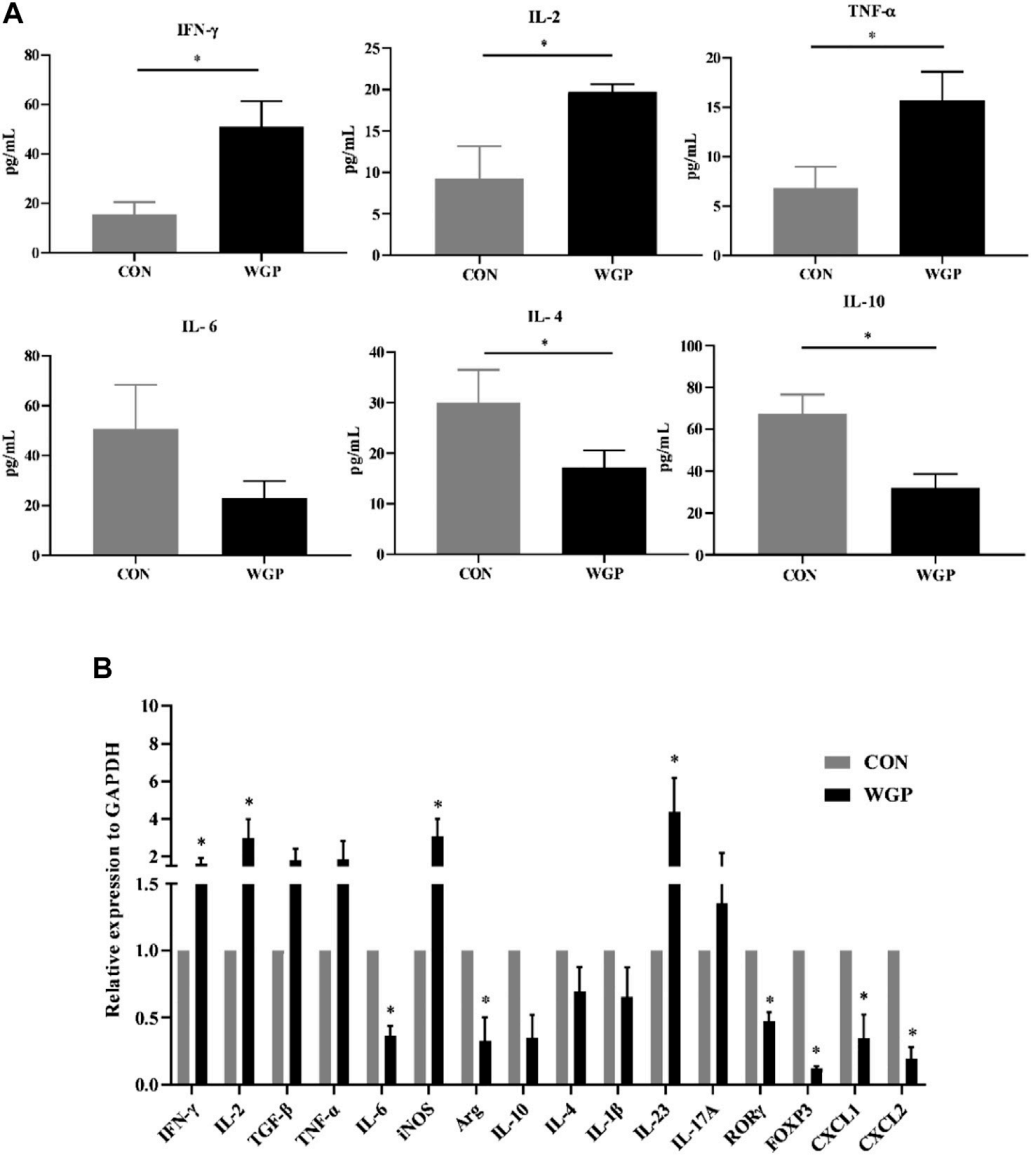
WGP alters the production of inflammatory cytokines and chemokines **(A)** After WGP treatment, multiple inflammatory cytokines were detected in the serum of AOM/DSS-treated mice by flow cytometry. **(B)** The mRNA expression of immune response-related molecules in the TME was determined by quantitative reverse transcription-PCR and calculated by the 2^−ΔΔCt^ method (*n* = 5–6). Representative results from three independent experiments are shown. Data represent the mean ± SEM. Statistical significance was determined by two-sided unpaired Student’s t-test. *p < .05.

With the changes in immune cells in the tumor microenvironment, the expression of cytokines has been further investigated. We measured the levels of inflammatory cytokines in serum, and the data showed that serum IFN-γ, IL-2, and TNF-α secretion increased in the WGP-treated mice, while IL-4 and IL-10 decreased. Other inflammatory cytokines, such as IL-6, showed no change (Figure 3A). Next, we examined whether the mRNA levels of cytokines in the tumor tissue were altered between water-treated and WGP-treated mice. Total mRNA was extracted from tumor tissue for RT-PCR. As shown in Figure 3B, IFN-γ, IL-2, iNOS and IL-23 were significantly increased, while IL-6, Arg-1, ROR-γt, and FOXP3 were significantly decreased in the TME of WGP-treated mice. Moreover, WGP inhibited the expression of the chemokines CXCL1 and CXCL2, which are related to angiogenesis and dysplasia-carcinoma transition processes in colon cancers (Jamieson et al., 2012; Katoh et al., 2013). The changes in these cytokines are related to the changes in infiltrating immune cells, which interact with each other to exert inhibitory effects during the development of CAC by WGP treatment.

### WGP protected against DSS-induced chronic colitis in WT mice

**FIGURE 4:**
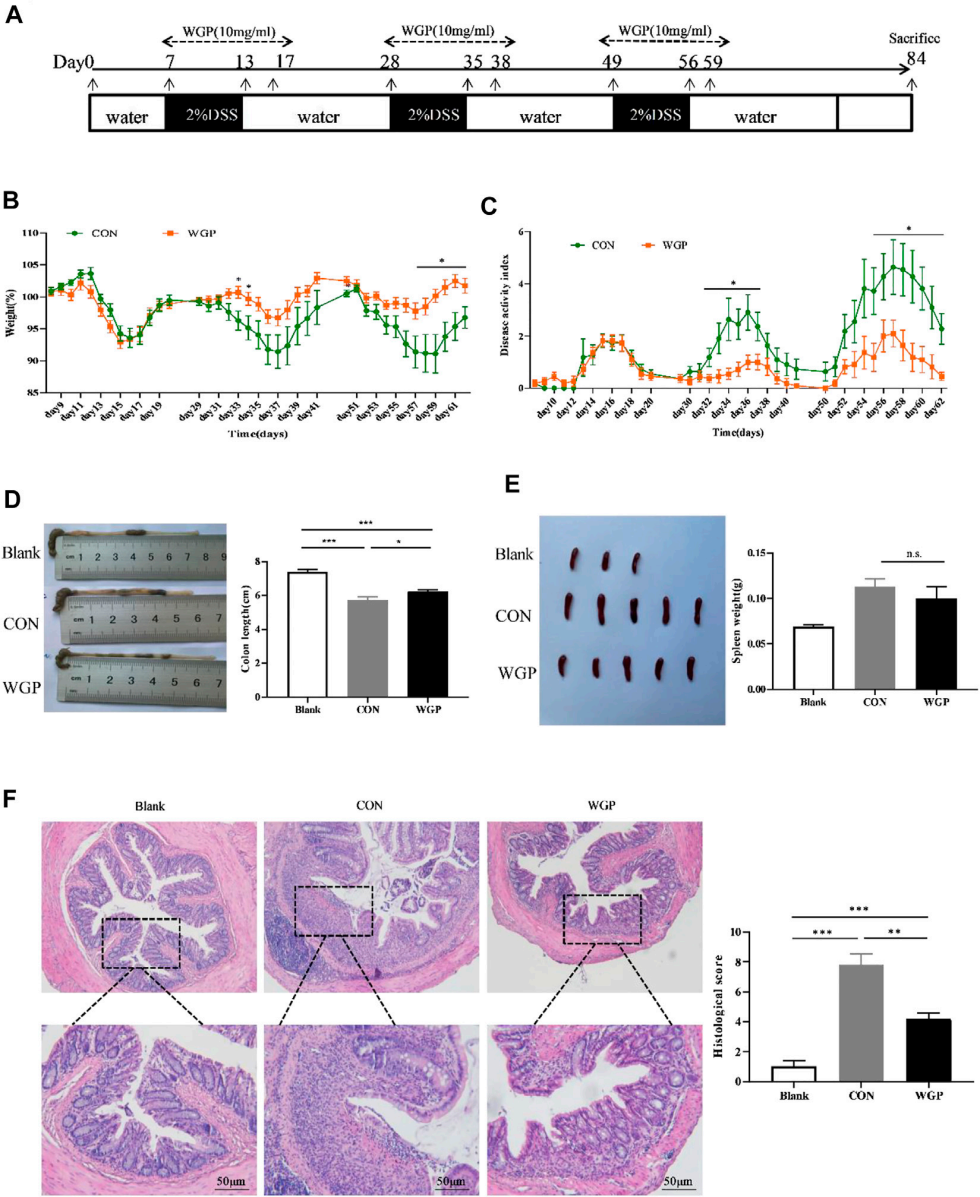
WGP prevented DSS-induced chronic colitis in C57BL/6 mice. **(A)** The experimental design of the therapeutic treatment with WGP on DSS-induced chronic colitis in C57BL/6 mice. **(B,C)** Body weight **(B)** and disease activity index**(C)** of mice that received 2% DSS-containing water (CON group, green dotted line) or 2% DSS combined with oral WGP (WGP group, orange dotted line). **(D–F)** Colon length **(D)**, spleen weight **(E)**, representative H&E image of distal colon sections and corresponding histological scores **(F)** at Day 84 after DSS exposure. Data are representative of three independent experiments with *n* = 10 mice per group. The statistical significance of differences was determined by multiple t tests **(B,C)** and one-way ANOVA **(D–F)**. *p < .05, **p < .01, ***p < .001, n. s. indicates no significant difference. The results are presented as the means ± SEMs.

This study showed that WGP has a protective effect on CAC; therefore, we hypothesized that WGP may affect the development of CAC during the chronic inflammation stage. To further test the role of WGP in inflammation-driven tumorigenesis, a model of chronic inflammation induced by DSS was evaluated. The therapeutic regimen of WGP in inflammation is consistent with that of CAC (Figure 4A). By Day 84, the results showed that WGP treatment markedly improved the clinical symptoms, WGP-treated mice lost less weight than CON mice (Figure 4B), and CON mice that received water treatment exhibited much more severe colitis symptoms, including severe mental malaise, loose or bloody stools that stuck to the anus, abundant bloody diarrhea, and a higher DAI score than those who received WGP treatment (Figure 4C). Moreover, other signs of colitis and colon length were significantly increased after WGP treatment (Figure 4D), although the change in the spleen was not obvious (Figure 4E). A detailed histological analysis consistent with the gross findings showed that crypt destruction, architectural and cytological atypia, and massive inflammatory cell infiltration were more aggressive and widespread in CON-treated mice than in WGP-treated mice (Figure 4F). According to the scoring criteria of intestinal mucosal injury, the histological score of the CON group was significantly higher than that of the WGP group, suggesting that WGP could protect the colonic tissue structure damage caused by chronic colitis. However, WGP did not significantly improve clinical signals such as body weight, DAI, colon length, spleen weight, or histological score in dectin-1^−/−^ mice with induced colitis (Supplementary Figure S3). In brief, these results suggest that WGP has a protective effect on DSS-induced chronic inflammation that is dependent on dectin-1.

### WGP alters the accumulation of immune cells in the cLP

**FIGURE 5:**
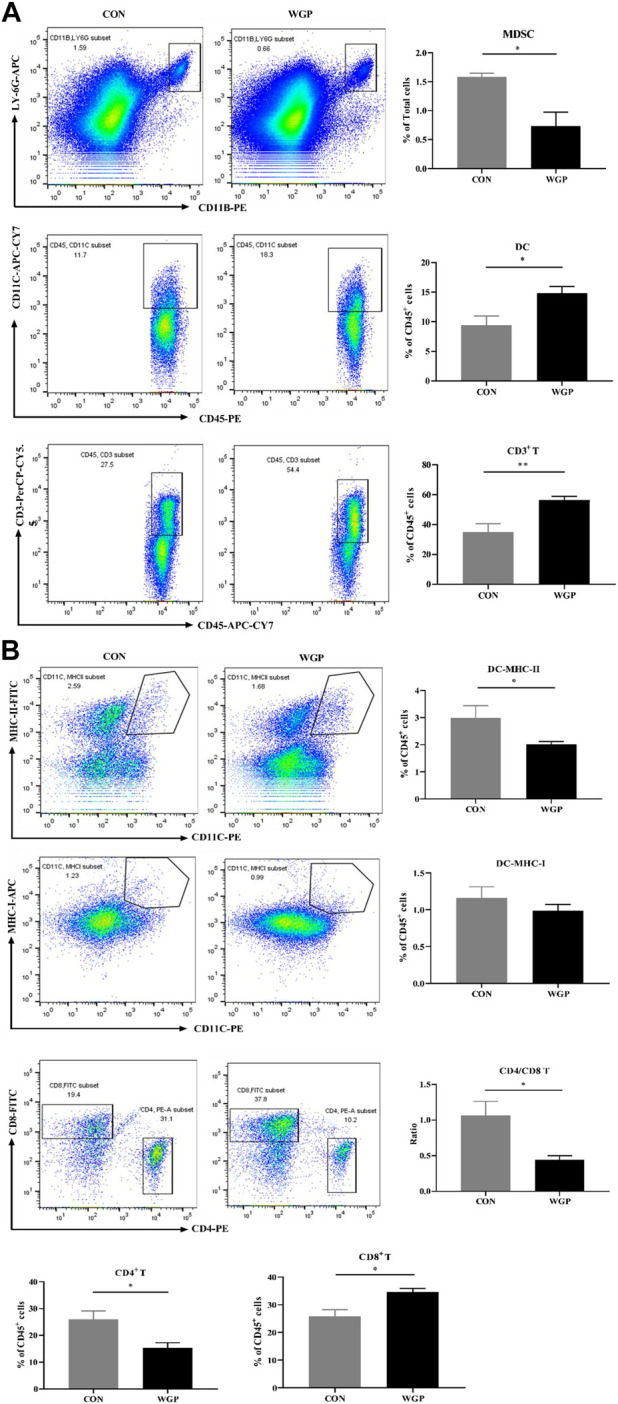
WGP-treated mice with DSS-induced chronic colitis were analyzed by flow cytometry **(A)** The frequency of MDSCs (CD11b^+^ LY-6G^+^), DCs (CD45^+^ CD11c^+^), and T cells (CD45^+^ CD3^+^) in the cLP after administration of DSS. **(B)** The percentages of DC and T-cell subtypes. Dot plots are gated on CD45^+^ cells. Data indicate the mean ± SEM of each group (*n* = 4–5) obtained from three independent experiments. Statistically significant differences are shown. *p < 0 05, **p < 0 01, ***p < 0,001, n. s. means no significant difference.

Histological evaluation of inflammatory cell infiltration into the colon revealed that CON mice experienced higher immune infiltration than WGP-treated mice during DSS treatment (Figure 4F). Subsequent analysis by flow cytometry showed that the infiltration of MDSCs, DCs, and CD3^+^ T cells in the lamina propria of the colon (cLP) was remarkably different. As shown in Figure 5A, DSS-treated colons from WGP-treated mice had dramatically reduced accumulation of MDSCs, but DC and CD3^+^ T cells in the cLP were significantly increased. We also examined the subsets of DCs and T cells. The results showed that the significant increase in CD3^+^ T cells may be due to the large increase in CD8^+^ T cells; although CD4^+^ T cells decreased greatly, the ratio of CD4/CD8 also decreased significantly. At the same time, there were no differences in the accumulation of DCs expressing MHC-I, but MHC-II + DCs were significantly decreased (Figure 5B). Similarly, in the DSS-induced colitis tissue of dectin-1^−/−^ mice, WGP treatment did not affect the infiltration of DCs and T cells, but MDSCs were still significantly reduced (Supplementary Figure S4). In addition, the data showed that WGP treatment did not affect the proportion of CD44^high^ CD62L^−^ T cells on CD3^+^ and CD8^+^ T cells, suggesting that WGP could not maintain the long-term existence of memory T cells and may not have the effect of preventing inflammation, which has certain significance for guiding the medication regimen of WGP (Supplementary Figure S5). These data indicate that WGP affects immune cells infiltration during the development of colitis, allowing increased DC and T-cell infiltration.

### WGP affects the production of cytokines in DSS-induced chronic colitis

**FIGURE 6:**
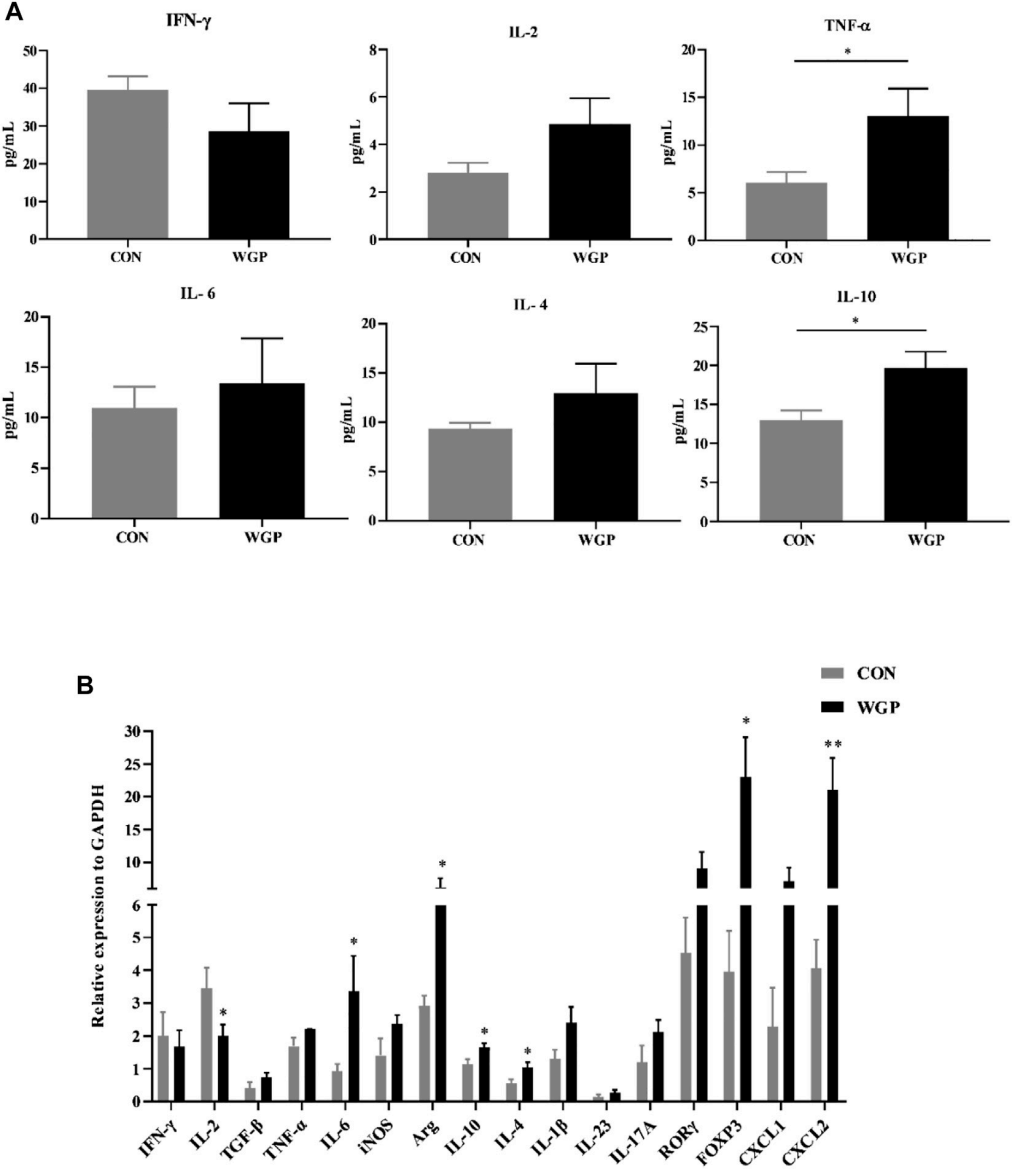
WGP affects the changes in inflammatory cytokines in chronic colitis **(A)** Multiple inflammatory cytokines were detected in the serum of DSS-treated mice by flow cytometry. **(B)**The mRNA expression of immune response-related molecules in the cLP was determined by quantitative reverse transcription-PCR and calculated by the 2^−ΔΔCt^ method. Representative results from three independent experiments are shown. Data represent the mean ± SEM (*n* = 4–8). Statistical significance was determined by unpaired Student’s *t*-test. *p < 0 05, **p < 0 01.

To evaluate the effect of WGP on cytokine production induced by immune cell changes, we determined the presence of inflammation-related cytokines by flow cytometry and RT-PCR. As shown in Figure 6, the serum levels of IL-10 and TNF-α in the WGP group were significantly increased (Figure 6A). The mRNA expression levels of IL-4, IL-6, and IL-10 in colons were increased following treatment with WGP, while IL-2 was decreased according to RT-PCR (Figure 6B). In addition, the expression levels of Arg-1, FOXP3, and CXCL2 in the WGP-treated group were higher than those in the group treated with water. These results indicate that WGP can regulate inflammatory cytokine production in mice with DSS-induced colitis.

## Discussion

Inflammation, although necessary for damage repair and fighting infection, can greatly affect proliferation, resistance to apoptosis, and cell transformation, thereby promoting tumor formation (Elinav et al., 2013). Most types of inflammation accompany and promote the occurrence of tumors (Fan et al., 2013), and chronic inflammation is now considered a possible feature of cancer (Greten and Grivennikov, 2019). As a special type of colorectal cancer, chronic inflammation is one of the main reasons for the formation of CAC. Our data showed that WT mice with CAC successfully established by AOM/DSS developed typical tumors at the end of the colorectal, with moderate or severe dysplasia of most epithelium in the tissues, irregular arrangement of crypts, and obvious infiltration of inflammatory cells in the mucosa. Notably, oral WGP treatment significantly reduced the severity of glandular hyperplasia and inflammatory cell infiltration and extended overall survival (Figure 1). In addition, DSS was used to simulate the chronic inflammation stage before CAC formation. The results also showed that WGP could slow down weight loss, improve DAI score, prolong colon length and reduce tissue damage, indicating that WGP could alleviate chronic colitis (Figure 4). This may suggest that WGP could be a novel approach for the prevention and treatment of colorectal cancer by protecting against chronic inflammation and thereby mitigating tumor development.

We next sought to delineate the mechanisms by which WGP confers its protection against CRC in this model. Currently, studies on the mechanism by which the AOM/DSS model induces CAC have focused on the influence of changes in immune cells and cytokines in the immune microenvironment (Elinav et al., 2013; Hirano et al., 2020). The intestinal microenvironment is composed of a variety of multiple immune cells, including intestinal epithelial cells, dendritic cells (DCs), myeloid-derived suppressor cells (MDSCs), macrophages, neutrophils, and T lymphocytes (Atreya and Neurath, 2008). As a key barrier of antitumor immunity, many studies have found that there is a large number of MDSCs in tumor-bearing mice (Veglia et al., 2021). It is widely accepted that MDSCs have suppressive effects on both the innate immune response *via* NK cells and the adaptive immune response *via* T cells by direct cell-cell contact (Katoh et al., 2013). MDSCs have been considered an endogenous antagonist of immune system function in mucosal inflammation due to their immunosuppressive effect on CD8^+^ T cells (Wang et al., 2020). Arg-1 is required for the immunosuppressive effects of MDSCs on CD8^+^ T cell cytotoxicity. Increased expression of Arg-1 in colon tumor tissue significantly inhibited the secretion of IFN-γ and IL-2 by colon CD8^+^ T cells (Katoh et al., 2013). A striking finding of our study was the increased infiltration of MDSCs and up-regulation of Arg-1 activity in colon tumor tissues, while WGP treatment significantly reduced the number and function of MDSCs. In addition, the decrease in CXCL1 and CXCL2 in tumor tissue induced by WGP may be related to MDSCs. Both CXCL1 and CXCL2 are ligands of CXCR2, which promotes tumor growth and progression by inhibiting the cytotoxic activity of CD8^+^ T cells (Katoh et al., 2013). Loss of CXCR2 dramatically suppresses colitis-associated tumorigenesis by inhibiting the infiltration of MDSCs into the colonic mucosa and tumors (Katoh et al., 2013). These results suggest that WGP may inhibit the growth and progression of CAC by inducing Arg-1-mediated MDSCs. However, During the development of colitis, our results showed that after oral administration of WGP, MDSCs were not recruited into intestinal tissues in large quantities, and the number of infiltrated MDSCs was significantly reduced, but the expression of Arg-1 in intestinal tissues was significantly upregulated. Studies have shown that G-MDSC exosomes attenuate DSS-induced colitis by inhibiting Th1 cell proliferation and promoting Treg cell expansion, which is related to Arg-1 activity (Wang et al., 2016). Our results showed that the increased mRNA level of FOXP3 in colitis tissues may be associated with significantly upregulated Arg-1. WGP regulates the immune suppression of G-MDSCs by inhibiting NFIA expression, and the effect of WGP on NFIA expression is dependent on C-Jun through the Dectin-1 pathway (Tian et al., 2015). However, in the CAC or chronic colitis microenvironment of dectin-1^−/−^ mice, changes in MDSCs induced by WGP treatment did not affect disease progression, suggesting that MDSCs do not dependent on the action of dectin-1.

On the other hand, DCs are specialized antigen presenting cells (APCs), which play a key role in inducing antitumor cytotoxic immune responses and are the core of the link between adaptive and innate immune systems (Wculek et al., 2020). Stimulation with WGP led to DC maturation and cytokine secretion, promoted CD4^+^ T cells to differentiate into Th1 cells, and induced tumor-specific CTLs (Ning et al., 2016). We also found that WGP had similar effects on human monocyte-derived DCs and induced CD8^+^ IFN-γ^+^ T-cell immune responses through the PI3K/AKT pathway (Ding et al., 2015). DC defects are involved in various mechanisms of colorectal cancer immune escape. Down-regulation of Notch2 signaling in intestinal epithelial cells leads to impaired DC differentiation ability, a reduced number of mature DCs and reduced antigen presentation ability, which promotes the development of colon cancer (Wang et al., 2021). In the tissues of colon cancer patients, patients with more activated DC infiltration had a better prognosis and longer disease-free survival, which was also related to the colocalization of IRF7 on DCs and CD8^+^ T cells expressing granzyme B in different tumor regions contributing to the antitumor effect (Kiessler et al., 2021). Our results show that WGP induces a significant increase in DC during CAC formation, which further induces the activation of CD3^+^ T cells. Importantly, DC-mediated presentation of MHC class I (MHC-I)/peptide complex is a crucial first step in priming CTL response (Rodriguez-Cruz et al., 2011), and the increase of MHC-I^+^ DC may be the main reason for the large increase of CD8^+^ T cell. In addition, WGP promoted the increase of Th1-type cytokines IFN-γ and IL-2 and the decrease of Th2-type cytokines IL-4 and IL-10, which can induce the differentiation of T cells, further demonstrated its anti-tumor ability. The decreased mRNA expression of FOPX3 and ROR-γt in tumor tissues may be related to the decrease of the total number of CD4^+^ T cells in tumor microenvironment. WGP can be recognized by DCs expressing dectin-1. Deficiency of the dectin-1 receptor completely abrogated WGP-mediated anti-tumor effects (Qi et al., 2011) and it is not surprising that the antitumor immune effect of DCs was eliminated in the tumor and inflammatory microenvironment of dectin-1^−/−^ mice. These results suggest that WGP alleviates CAC mainly by Dectin-1-dependent DC cells.

In DSS-induced colitis, oral administration of β-glucan may both aggravate intestinal inflammation by increasing levels of inflammatory cytokines and chemokines in the colon and inhibit the expression of pro-inflammatory cytokines, exhibiting an anti-inflammatory function against colitis (Heinsbroek et al., 2015; Liu et al., 2015). Some studies suggest that WGP was able to revert LPS- and IL-4/IL-13-mediated macrophage responses and reduce the release of pro-inflammatory cytokines (Aguzzi et al., 2020). Other studies have shown that WGP enhances the pro-inflammatory response by enhancing the energy state of macrophages in C. krusei-treated bilateral nephrectomy mice (Issara-Amphorn et al., 2021). In our results, WGP inhibited DSS-induced chronic colitis and maintained intestinal homeostasis mainly by inhibiting tumor-oriented DCs.

DCs derived from healthy intestinal lamina propria (LP) represent an immature phenotype characterized by low-level expression of costimulatory cytokines (Strauch et al., 2010). Accumulation of DCs in lamina of IBD and DSS colitis can prevent colitis (Zhang et al., 2021). LP-DCs continuously take up antigens from the intestine and migrate to the draining mesenteric lymph nodes (MLNs), where they present antigens to T cells to further drive different T-cell fates (Coombes and Powrie, 2008). DC with low expression of MHC-II, CD86 and CD80 and high expression of IL-10 can improve colonic inflammation and reduce local lesion injury, which is related to the decrease of CD4^+^ T cell number in the colon and the differentiation of naive T cells into Foxp3 Treg cells (Jin et al., 2019; Okada et al., 2021; Rao et al., 2022). Our results showed that in the DSS-induced inflammatory microenvironment, the proportion of infiltrating DCs and the reduction of MHC-II^+^ expressing DCs in the colon of WGP-treated mice were significantly increased compared with CON mice. The decrease of CD4^+^ T cells and the mRNA of Foxp3 expression up-regulation in colon tissues suggest that WGP promotes the differentiation of T cells into Treg cells. The up-regulation of IL-4 mRNA and down-regulation of IL-2 in inflammatory tissues also suggest that WGP-induced differentiation of T cells alleviates inflammation.

On the other hand, LP-DCs can produce high levels of the immunosuppressive cytokine IL-10, whose genetic defect causes gastrointestinal inflammation in humans and mice (Hagenlocher et al., 2019), and its induced antitumor effect is mainly dependent on CD8^+^ T cells (Qiao et al., 2019). C. krrusei β-glucan stimulation of BMDCs increased IL-10 production by T cells and also increased IL-10 production by BMDCs (Dinh et al., 2020), indicating that IL-10 production was co-mediated by TLR2 and Dectin-1. Our results also expressed a similar view that WGP promoted high IL-10 expression in association with increased DC and CD8^+^ T cells. When dectin-1 is lost, WGP has no therapeutic effect on DSS-induced chronic colitis. Therefore, it can be speculated that IL-10-mediated DC may be the main reason why WGP inhibits chronic colitis. In conclusion, with the development of chronic inflammation in tumors, the change in the immune microenvironment gradually transforms tolerant DCs into antitumor DCs, which may be an important reason why WGP inhibits CAC by inhibiting intestinal inflammation.

In addition, WGP plays an important role in the regulation of cytokines associated with colitis. IL-6 has a unique tissue-protective effect on gastrointestinal mucosa, which is necessary for tissue regeneration after injury (Kuhn et al., 2014). Administration of recombinant IL-6 to mice lacking EGFR in myeloid cells given DSS protected them from weight loss and restored epithelial proliferation and STAT3 activation compared with administration of DSS alone (Srivatsa et al., 2017). These considerations are further supported by the result that IL-6 reduces STAT3 expression in intestinal epithelial cells (IECs) and increases the severity of DSS-induced colitis in IL-6^−/−^ mice (Grivennikov et al., 2009). However, when chronic inflammation gradually develops into tumors, that is, in the early stage of CAC formation, IL-6 up-regulates and activates STAT3, stimulates the malignant proliferation of IECs, and promotes the occurrence of tumors by inhibiting the activities of DCs and antitumor cytotoxic CD8^+^ T cells and inducing the transformation of M1-type TAMs to M2 (Tsukamoto et al., 2018), further promoting tumorigenesis. According to the changes in the immune microenvironment, the down-regulation of IL-6 secretion by WGP slowed tumorigenesis. Other pro-inflammatory factors, such as TNF-α, are also important for the development of colitis. WGP induces mature DCs to secrete high levels of TNF-α and IL-12, further eliciting adaptive immune antitumor effects (Ning et al., 2016). In the inflammatory response, inhibition of TNF-α expression in serum can reduce colitis (Xiao et al., 2016; Koelink et al., 2020), and these changes also indicate that TNF-α plays an important role in the development of colitis to colon tumors. Although our data showed that WGP increased TNF-α expression in colon tissues during the chronic inflammatory phase, it may be related to the transmission in Treg cells and CD8^+^ T cells with increased TNF-αR2 signaling (Punit et al., 2015).

Taking these ideas into account, we believe that the inhibition or treatment of molecules with multiple regulatory inflammatory pathogenic factors will have a higher probability of success in the fight against cancer. As a multifunctional immunomodulator, WGP has a positive effect on both immune cells and cytokines. Our results show that WGP has a good therapeutic effect on chronic colitis and CAC and is expected to become a new drug for the prevention and treatment of colorectal cancer.

## Data Availability

The original contributions presented in the study are included in the article/Supplementary Material, further inquiries can be directed to the corresponding authors.

## Abbreviations

AOM, Azoxmethane; APC, Antigen presenting cell; BMDM, Bone marrow-derived macrophages; CAC, Colitis-associated colorectal cancer; CD, Crohn’s disease; CLP, Colonic lamina propria; CRC, Colorectal cancer; CRI, Cancer-associated inflammation; DAI, Disease activity index; DC, Dendritic cell; DSS, Dextran sulfate sodium; FBS, Fetal bovine serum; IBD, Inflammatory bowel disease; IEC, Intestinal epithelial cell; MDSC, Myeloid-derived suppressor cell; MLN, Mesenteric lymph node; UC, Ulcerative colitis; WGP, Whole β-glucan particle.
