# Supplementary material for: Whole β-glucan particle attenuates AOM/DSS-induced colorectal tumorigenesis in mice via inhibition of intestinal inflammation
Source: Front Pharmacol. 2023 Jan 12;14:1017475. doi: 10.3389/fphar.2023.1017475 (PMC9877317; doi:10.3389/fphar.2023.1017475)

**Supplementary Information**

**β-glucan attenuates AOM/DSS-induced colorectal tumorigenesis in mice via inhibition of intestinal inflammation**

Xie et al

**Table 1: Primer sequences for qPCR**

Gene Forward Reverse
Foxp3 AGGAGCCGCAAGCTAAAAGC TGCCTTCGTGCCCACTGT
ROR-*γ*t CCACTGCATTCCCAGTTTCT CGTAGAAGGTCCTCCAGTCG

IL-6 GAGAAAGAGTTGTGCAATGGC CCAGTTTGGTAGCATCCATCAT

IL-17 ATGGAAGAGTATGAGCGGAACCT GGGTCGTGGTTGATGCTGTAG

IFN-γ CGGCACAGTCATTGAAAGCC TGCATCCTTTTTCGCCTTGC

INOS CCAAGCCCTCACCTACTTCC CTCTGAGGGCTGACACAAGG

Arg-1 CAGAAGAATGGAAGAGTCAG CAGATATGCAGGGAGTCACC

IL-1β CAGGCAGTATCACTCATTGTGG GTGCAGTTGTCTAATGGGAACG

TGF-β TGCTAATGGTGGACCGCAA CACTGCTTCCCGAATGTCTGA

TNF-α TGTAGCCCACGTCGTAGCAAA CTGGCACCACTAGTTGGTTGT

IL-10 ACTGGCATGAGGATCAGCAG CTCCTTGATTTCTGGGCCAT

IL-4 GGTCTCAACCCCCAGCTAGT CCCTTCTCCTGTGACCTCGT

IL-2 AAGTGTGGGAAAACGGGGTG GAGCACAAATGTCTCCGTCA

IL-23 TCAGACAGGTTCCAGCCCTA CCCCAATCGCCTTGATCTCT

CXCL1 CTGTGCTAGTAGAAGGGTGTTG ACCAGGAGAAACAGGGTTAAAG

CXCL2 GCGGTCAAAAAGTTTGCCTTGA AGGCTCCTCCTTTCCAGGTC

GAPDH TGACCACAGTCCATGCCATC GACGGACACATTGGGGGTAG

**Supplemental Fig. 1** Effect of WGPon AOM/DSS-induced CAC in Dectin-1^-/-^ mice

**The** CAC induced by AOM/DSS in dectin-1-/- mice and the WGP administration regimen were similar to those in WT mice. **(A-B)** Body weight (A) and disease activity index (B) during each treatment cycle of mice that received 2% DSS-containing water (CON) group, green dotted line), or 2% DSS combined with WGP oral (WGP group, orange dotted line). For statistical comparisons, an asterisk indicates CON vs. WGP. **(C-E)** Colon length (C), spleen weight (D), and the number of tumor representative pictures of colons and the size distribution of colorectal tumors (E) at Day 84 in AOM/DSSinduced mice treated with or without WGP. **(F)** Representative H&E staining of distal colon cross-sectionsafter DSS exposure. Data are representative of three independent experiments with n=5 mice per group. The statistical significance of differences was determined by multiple t tests (A, B) and Student’s unpaired t test (C, D). **P*< 0.05, ***P* < 0.01. The results are presented as the means ± SEMs.


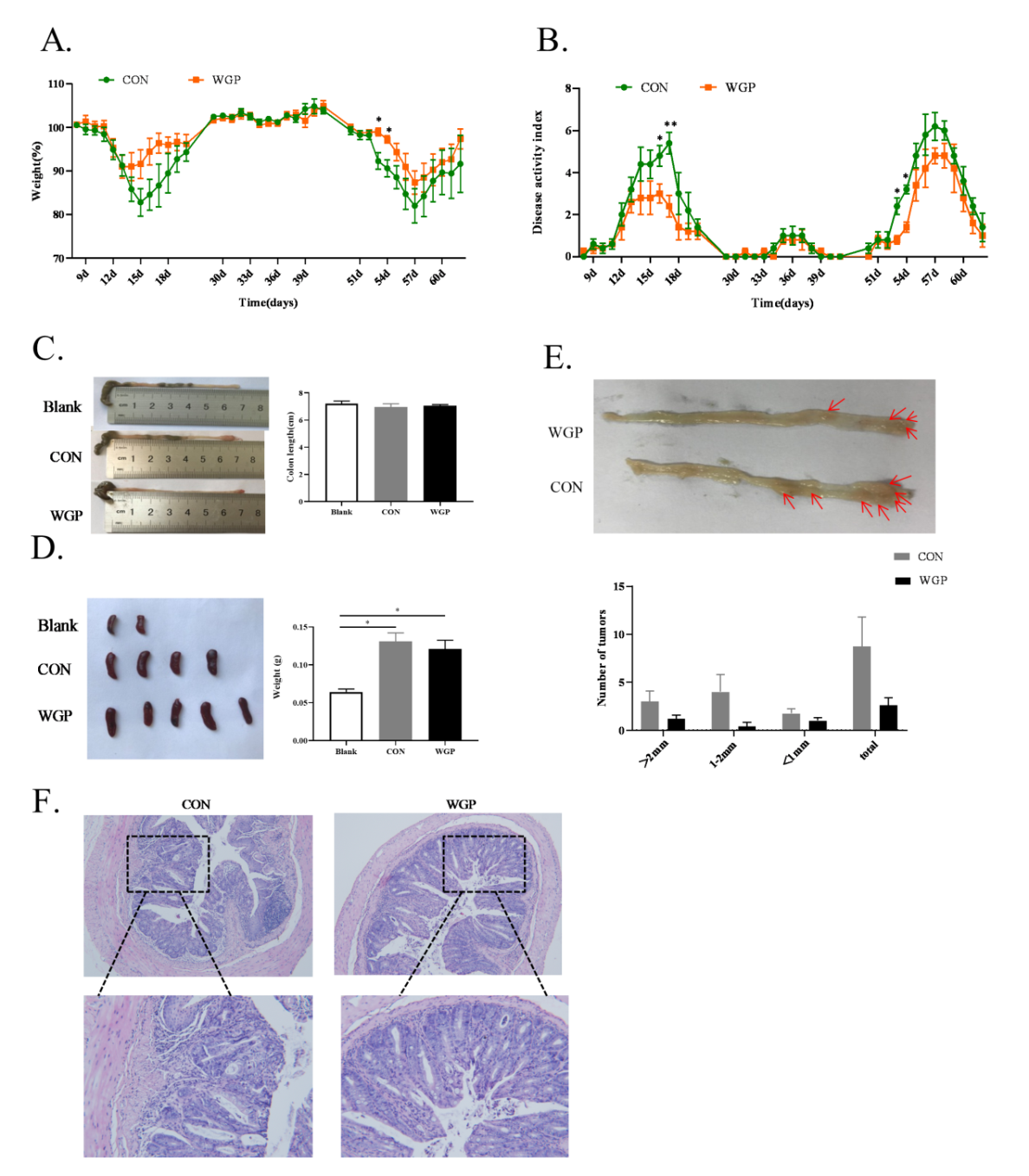


**Supplemental Fig. 2** Immune cell infiltration in Dectin-1^-/-^ mice with CAC

At Day 84 , the representative staining, percentage of MDSCs (CD11b^+^LY-6G^+^), macrophages (CD11b^+^F4/80^+^)gated in total cells, dendritic cells (DCs) (CD45^+^CD11c^+^), and T cells (CD45^+^CD3^+^) gated in CD45^+^ cells of the tumor were determined by flow cytometry. Numbers adjacent to the outlined areas indicate the percentage of the gated population in each group (n=3-5). **P*<0.05vs. CON by unpaired Student’s t test.The results are presented as the means ± SEMs.


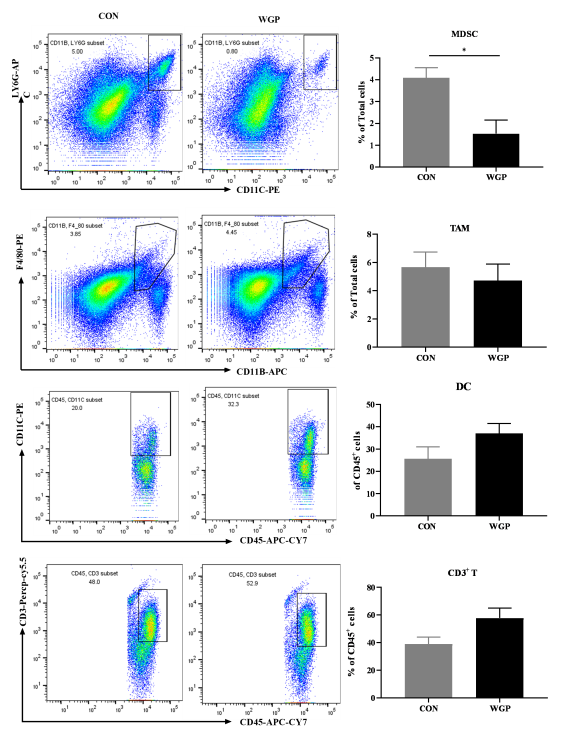


**Supplemental Fig. 3** Effect of WGP on DSS-induced chronic colitis in Dectin-1^-/-^ mice

The experimental design of the therapeutic treatment with WGP on DSS-induced chronic colitis in Dectin-1^-/-^ mice was similar to that in WT mice. **(A, B)** Body weight (A) and disease activity index(B) of mice that received 2% DSS-containing water (CON group, green dotted line) or 2% DSS combined with WGP orally (WGP group, orange dotted line). **(C-E)** Colon length (C), spleen weight (D), representative H&E image of distal colon sections and corresponding histological scores (E) at Day 84 after DSS exposure. Data are representative of three independent experiments with n=10 mice per group. The statistical significance of differences was determined by multiple t tests (A, B) and unpaired t tests (C-E). **P*< 0.05, ***P*< 0.01, ****P*< 0.001. The results are presented as the means ± SEMs.


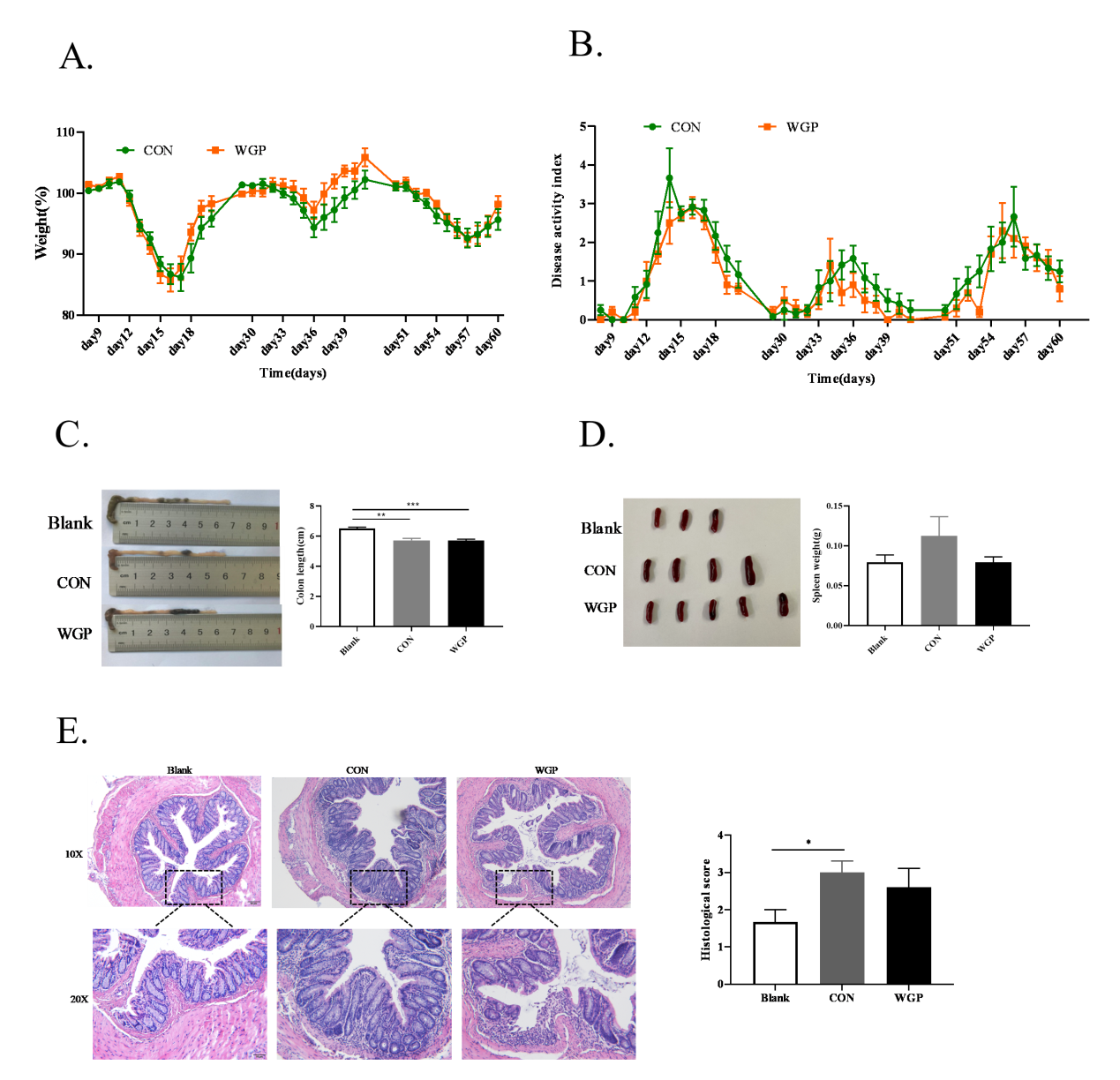


**Supplemental Fig. 4** Infiltration of immune cells in DSS-treated Dectin-1^-/-^ mice with chronic colitis

The frequency of MDSCs (CD11b^+^LY-6G^+^), DCs (CD45^+^CD11c^+^), and T cells (CD45^+^CD3^+^) in the cLP after administration of DSS. Data indicate the mean ± SEM of each group (n=5) obtained from three independent experiments. Statistically significant differences are shown.∗∗*p* < 0.01, ∗∗∗*p* < 0.001by unpaired Student’s t test.


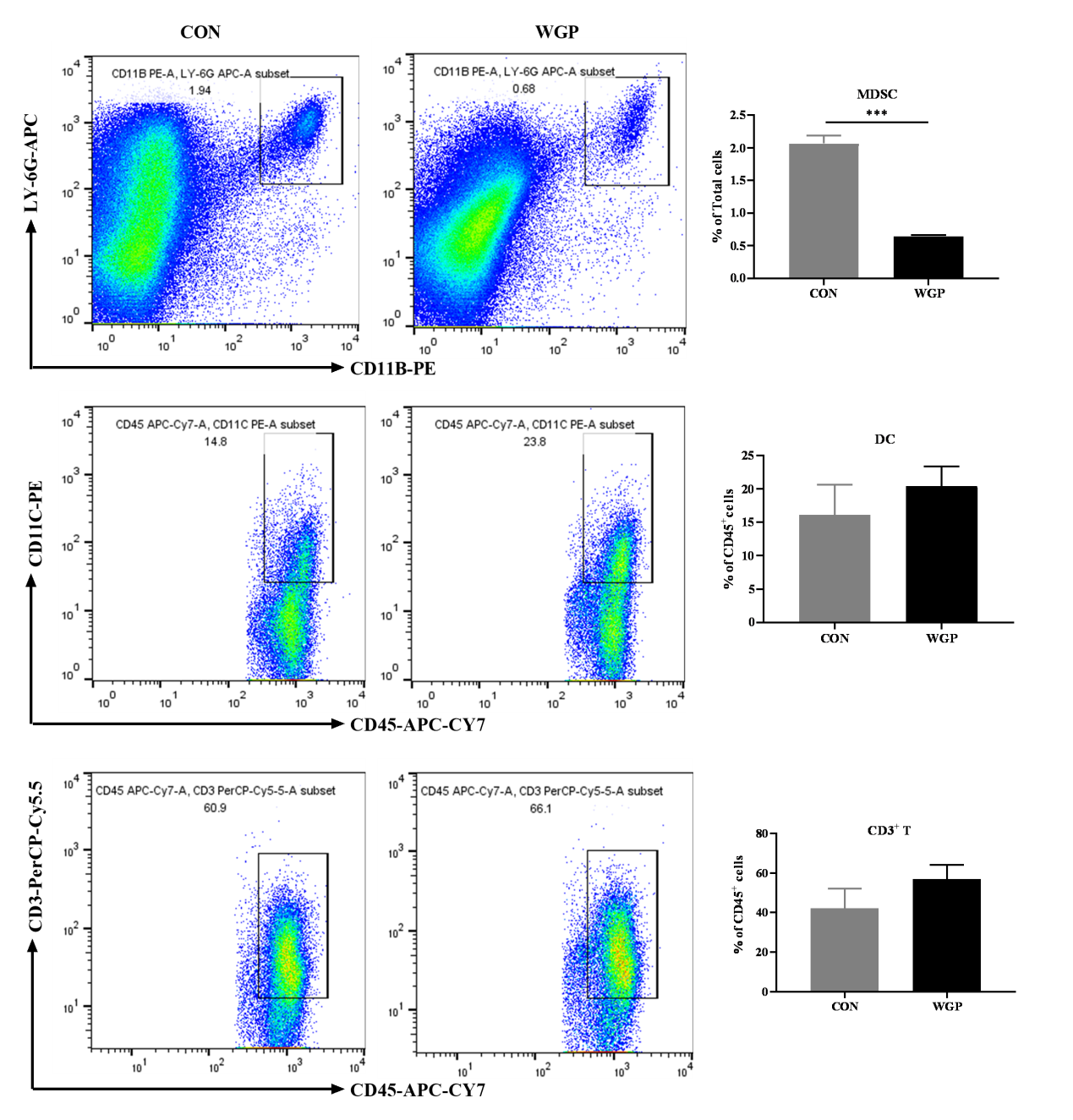


**Supplemental Fig. 5** Preventive effect of WGP on DSS-induced chronic colitis in WT mice

Flow cytometry was used to examine the expression of memory T cells in colitis tissue after WGP treatment.The frequency ofmemory T cells (CD62 L^-^ CD44^high^) in the cLP after administration of DSS.Data indicate the mean ± SEM of each group (n=5) obtained from three independent experiments.


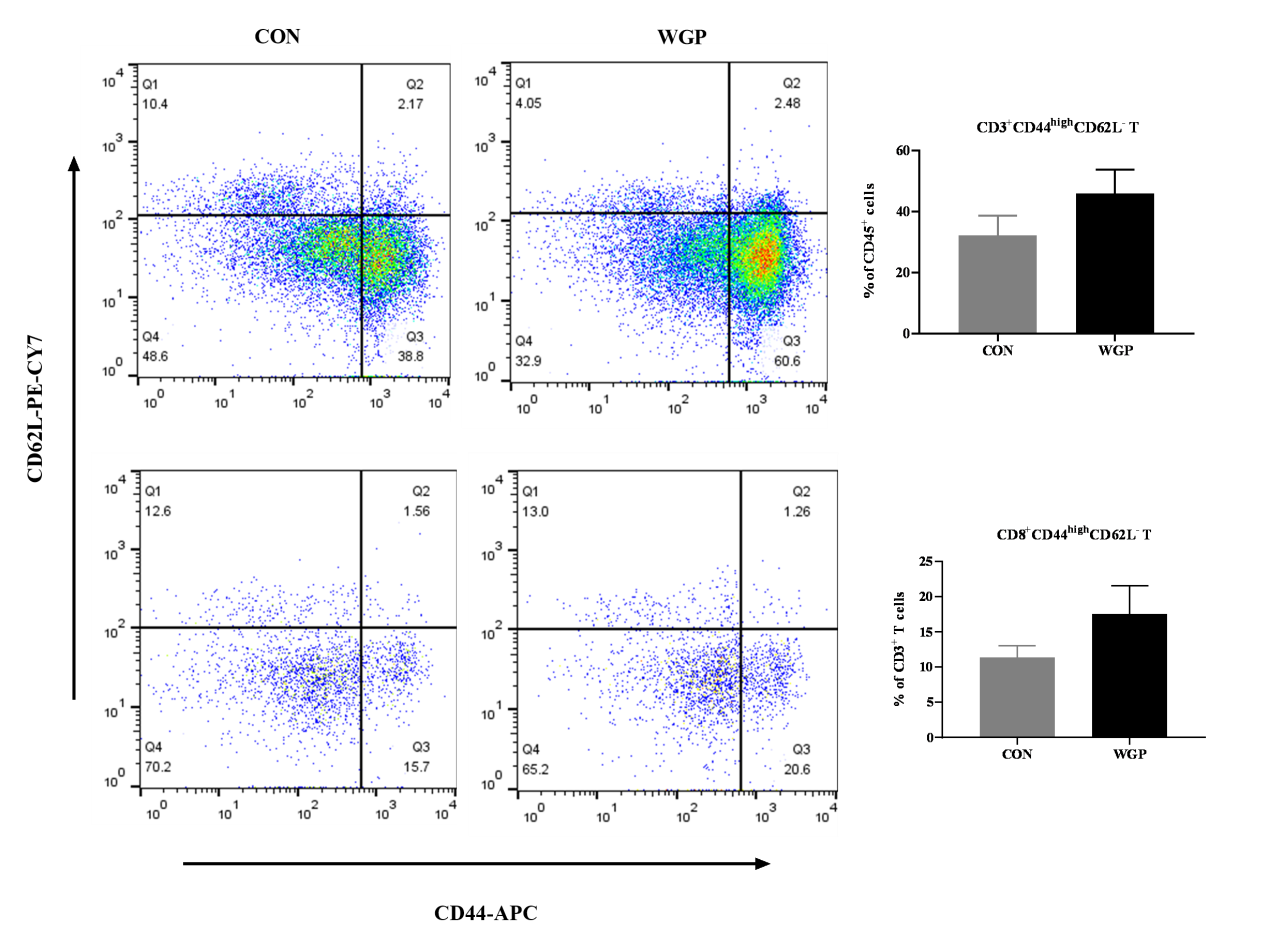

Supplement: Supplementary file 1 [file DataSheet1.docx]
